# Supplementary material for: Cellular advective-diffusion drives the emergence of bacterial surface colonization patterns and heterogeneity
Source: Nat Commun. 2019 Jun 6;10:2471. doi: 10.1038/s41467-019-10469-6 (PMC6554397; doi:10.1038/s41467-019-10469-6)
Supplement: Supplementary file 1 — Supplementary Information [file 41467_2019_10469_MOESM1_ESM.pdf]

Supplementary information

Rossy et al., *Cellular advective-diffusion drives the emergence of bacterial surface colonization patterns and heterogeneity*

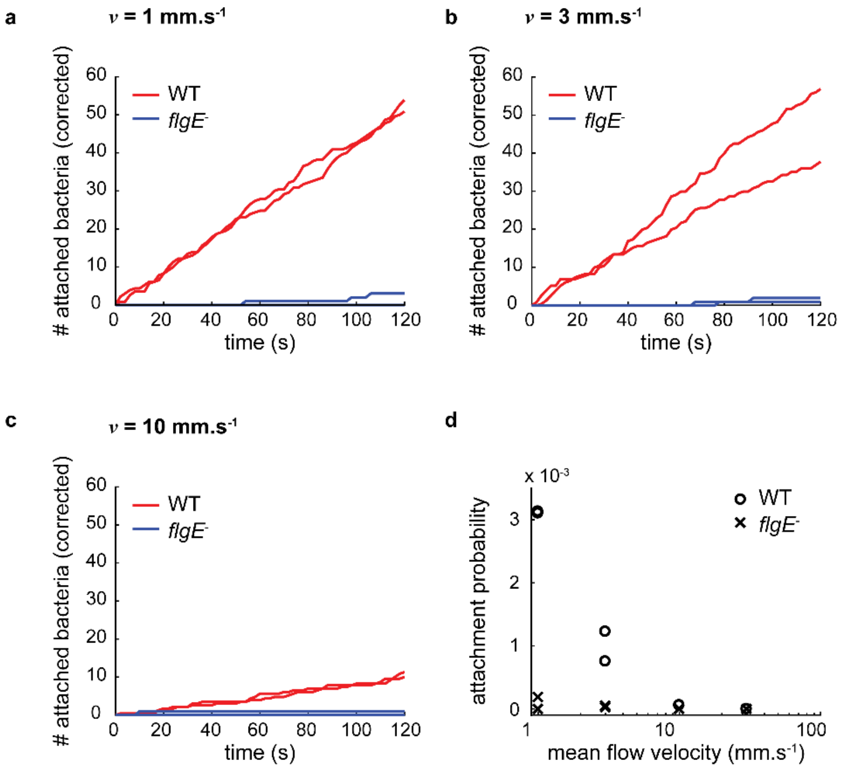

Supplementary Figure 1: Rate and probability of attachment as a function of flow speed for WT and *flgE*$^-$ mutants. (a-c) We quantified the number of swarmer *C. crescentus* cells (WT in red and *flgE*$^-$ in blue) that attached to the surface of a microchannel over 2 min at 1 mm. s^{-1} **(a)**, 3 mm.s^{-1} **(b)** and 10 mm.s^{-1} **(c)** mean flow velocity. We performed these experiments with a mixture of WT expressing mKate and *flgE*$^-$ expressing Venus. To normalize the fluxes of incoming bacteria, we divided the number WT cells by the relative abundance of WT to *flgE*$^-$ cells. **(d)** Probability of attachment of WT and *flgE*$^-$ cells as a function of flow velocity. As flow intensity increases, the colonization advantage provided by swimming motility vanishes.

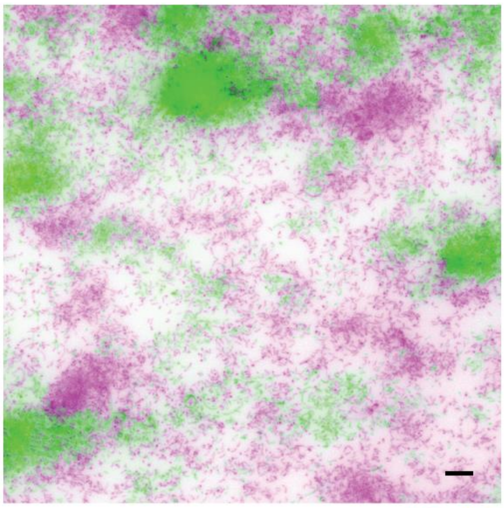

Supplementary Figure 2: Competition for surface colonization between WT and *flgE*$^{-}$ cells. A mixed population of *C. crescentus* WT (magenta) and *flgE*$^{-}$ (green) was initially loaded in the microchannel and grown for 24h under intermediate flow (7 mm.s^{-1}). While both WT and mutant colonize the surface in the form of microcolony clusters, the WT strain colonized a larger proportion of the surface with single isolated cells. This allows WT to invade green *flgE*$^{-}$ microcolonies. *flgE*$^{-}$ cells colonize a smaller proportion of the surface, mostly as large clonal clusters, but are unlikely to invade WT biofilms.
