# Supplementary material for: Cellular advective-diffusion drives the emergence of bacterial surface colonization patterns and heterogeneity
Source: Nat Commun. 2019 Jun 6;10:2471. doi: 10.1038/s41467-019-10469-6 (PMC6554397; doi:10.1038/s41467-019-10469-6)
Supplement: Supplementary file 2 — Description of Additional Supplementary Files [file 41467_2019_10469_MOESM2_ESM.pdf]

Description of Additional Supplementary Files

Supplementary Movie 1 – *C. crescentus* biofilm in strong flow: timelapse visualization of a *C. crescentus* biofilm growing in strong flow (mean flow velocity: 27 mm.s^{-1}) during 24h. Clonal clusters grow around the cells that were initially attached to the surface, while subsequent attachment of new bacteria is rare.

Supplementary Movie 2 – *C. crescentus* biofilm in weak flow: timelaspe visualization of a *C. crescentus* biofilm growing in weak flow (mean flow velocity: 2.5 mm.s^{-1}) during 24h. Single bacteria can frequently attach to the surface, thereby speeding up colonization and leading to the formation of patterns that are less patchy than in strong flow.
